# Epidemiology and burden of pediatric burns in underdeveloped minority areas in Guangxi, China from 2014 to 2020: a retrospective study

**DOI:** 10.3389/fpubh.2025.1566173

**Published:** 2025-06-04

**Authors:** Ziren Lin, Nahlah Abduljaleel Yahya Al-Saidi, Jiamei Lin, Yongfang Wu, Liuting Lan, Cheng Huang, Feiteng Liang, Zhiqun Huang

**Affiliations:** ^1^Department of Burn Surgery, Baise People's Hospital, Baise, Guangxi, China; ^2^Faculty of Medicine, MAHSA University, Kuala Lumpur, Malaysia; ^3^Department of Burn Surgery, Affiliated Hospital of Youjiang Medical University for Nationalities, Baise, Guangxi, China; ^4^Department of Pediatrics, Baise People's Hospital, Baise, Guangxi, China

**Keywords:** children, epidemiology, disease burden, associated factors, underdeveloped minority areas good health and well-being, clean water and sanitation, industry, innovation and infrastructure

## Abstract

**Background:**

Burns are a significant cause of accidental injuries in children worldwide. This study investigated the epidemiology and burden of pediatric burns to identify the associated factors with these injuries and to formulate prevention strategies in underdeveloped minority areas in Guangxi, China.

**Methods:**

A retrospective study was conducted on 660 pediatric burns admitted to two tertiary hospitals in Baise, Guangxi. Demographic data, burn characteristics, clinical factors, and burden information were analyzed. Multiple linear regression was performed to identify the associated factors associated with the length of hospital stay (LOHS) and costs.

**Results:**

660 pediatric burn cases were recorded from January 2014 to December2020. The median age of pediatric burn patients was 2 years. Winter is the peak period of pediatric burns. Scalding was the most frequent cause followed by flame burns. The median total body surface area (TBSA) affected being 6%. The median length of hospital stay (LOHS) was 9 days. The median cost was 7,558 CNY. The major associated factors for LOHS were the response rate [standardized beta coefficient (SBC) = 0.391], TBSA (SBC = 0.357), operations (SBC = 0.350). The major associated factors for cost were LOHS (SBC = 0.468), TBSA (SBC = 0.306), operations (SBC = 0.215), response rate (SBC = 0.120) and region (SBC = 0.081). The *p* value of all associated factors were *p* < 0.05.

**Conclusion:**

Scalds and flame burns are key targets for prevention among children under 3 years in underdeveloped minority areas of Guangxi, China. Winter is identified as the peak season for pediatric burns. The burden of pediatric burns is associated with multiple associated factors; these findings provide a foundation for assessing the epidemiology and burden of pediatric burns in this areas. Given the central role of caregivers in daily child supervision, addressing their knowledge, caregiving practices, and environmental awareness—alongside consideration of the broader contextual factors—can help foster a safer and more sustainable caregiving environment for children.

## Introduction

Despite modern advancements in burn treatment and care, which has significantly reduced the mortality rate among burn patients (1), burns remain the fourth most common and one of the deadliest types of injury worldwide (2). Burns pose a significant public health challenge, leading to substantial morbidity, disability and mortality, especially in developing nations. Annually, over 300,000 individuals succumb to burns, with many more are affected by scalds, electrocutions and chemical burns. This highlights a severe global public health concern, as noted by the World Health Organization (WHO) (3). As one of the world’s largest developing countries, China potentially has one of the highest numbers of pediatric burn cases globally (4). The incidence of burns varies significantly across different countries and within different regions of the same country (5).

While high-income countries have made significant progress in reducing burn-related deaths among children, the underdeveloped regions continue to bear a considerable burden of pediatric burn injuries and fatalities (6, 7). Factors such as poor economic conditions, lower education levels, mountainous and inaccessible terrain, isolated communities and inadequate transportation facilities contribute to this burden. Medical facilities are often sparse and vary significantly in the level of care that can be provided (8). This study aims to provide evidence for the serious consequences of pediatric burns in underdeveloped minority areas in Guangxi, China. This retrospective study was conducted at the department of Burn Surgery, Baise People’s Hospital, as well as the Affiliated Hospital of Youjiang Medical University for Nationalities between January 2014 and December 2020.

## Methods

### Ethical approval and data extraction

This retrospective study was approved by the Ethics Committee of Baise People’s Hospital (Ethics No.: KY2023121801). Data on pediatric burns (ages 0–12) were collected using the International Classification of Diseases, 10th Revision (ICD-10) (9), by searching the Healthcare Information System (10). The study included all pediatric burn patients admitted to Baise People’s Hospital and the Affiliated Hospital of Youjiang Medical University for Nationalities for scalding, flame-associated injuries and other accidental injuries which occurred between January 2014 and December 2020. The collected variables included demographic data, burn etiology, percentage of total body surface area (TBSA %), the presence of full-thickness burns, inhalation injuries, anatomical location of the burn injury, burn wound infection, surgeries, length of hospital stay (LOHS), patient outcomes and total medical costs upon discharge.

### Operational definitions

Outcomes: The outcomes were categorized as death, ineffective, improved and cured based on the healing of the burn wounds and the pediatric burn conditions at discharge. If the patient was deceased at discharge, the outcome was classified as “dead.” Classifications of ineffective, improved and cured were given to patients with the burn wound areas that did not significantly decrease or worsened, were not completely eliminated and were completely healed with no residual wounds after treatment, respectively. In clinical practice, medical workers hope that the outcome of burn children after standardized burn treatment is improve or curative. However, in underdeveloped minority areas, due to limitations in cultural and economic conditions, non-standard treatment approaches and traditional folk remedies remain in use. In this study, the response rate was defined as the proportion of pediatric burns whose guardians, after adequate communication and explanation of the disease course by medical personnel, agreed to continue receiving standardized burn treatment during hospitalization, particularly after the early phase when the children condition had stabilized. In contrast, the no response rate referred to the proportion of pediatric burns whose guardians, after the stabilization of the children condition, voluntarily requested discharge against medical advice due to financial constraints and/or a preference for non-standard or traditional folk treatments, thereby discontinuing standardized burn treatment.

Burn surgery: Burn surgery was defined as any surgical procedure performed under general or regional anesthesia in an operating room setting for the treatment of burn injuries. This included surgical wound debridement and/or escharectomy, split-thickness skin grafting, and local or regional flap reconstruction. Procedures such as bedside dressing changes, enzymatic debridement, or minor debridement without anesthesia in the ward were excluded from this definition. The decision to perform surgery was based on clinical indications such as deep partial-thickness or full-thickness burns, nonviable tissue requiring excision, or wounds unlikely to heal spontaneously. Timing of surgical intervention typically occurred within the first 3–7 days post-injury, in accordance with institutional burn care protocols.

Burn infection: The American Burn Association (ABA) consensus conference, convened in 2007, and it aimed to standardize the definitions of infections and sepsis in burn patients. According to the ABA, wound colonization is defined as a low concentration of bacteria on the wound surface, the absence of invasive infection, and fewer than 10^5 bacteria per gram of tissue. Local wound infection is characterized by a high concentration of bacteria in the wound or wound eschar, the absence of invasive infection, and more than 10^5 bacteria per gram of tissue. Invasive wound infection involves the presence of more than 10^5 pathogens per gram of tissue in the wound, invasion or destruction of unburned skin/tissue, with or without the presence of sepsis. Sepsis is identified as “a change in the burn patient that triggers concern for infection” and is specified by triggers that must be linked to a confirmed infection (11). Burn infection was defined as the presence of pathogenic microorganisms in the burn wound tissue that results in local or systemic signs of infection, requiring clinical intervention. Diagnostic criteria included one or more of the following: purulent wound exudate, change in wound color or odor, increasing eschar or necrosis, delayed wound healing, systemic inflammatory response (e.g., fever, leukocytosis), and/or positive microbiological cultures from wound swabs, tissue biopsies, or blood samples. Infection was distinguished from mere colonization by the presence of clinical signs of inflammation and/or histological evidence of microbial invasion into viable tissue. Burn wound infections were further classified as superficial wound infection, deep invasive wound infection, or burn wound sepsis, according to the extent of microbial invasion and systemic involvement.

Burn management (7, 12): Both Baise People’s Hospital and the Affiliated Hospital of Youjiang Medical University for Nationalities are tertiary hospitals in Guangxi, China. At these centers, the standard protocol for burn treatment encompasses several main aspects: first aid, fluid resuscitation, operation and wound dressing management, infection prevention and treatment, nutritional therapy, rehabilitation and the diagnosis and treatment of inhalation injuries. Additionally, the appropriate wound management is selected based on the specific cause of the burn.

**Figure 1:**
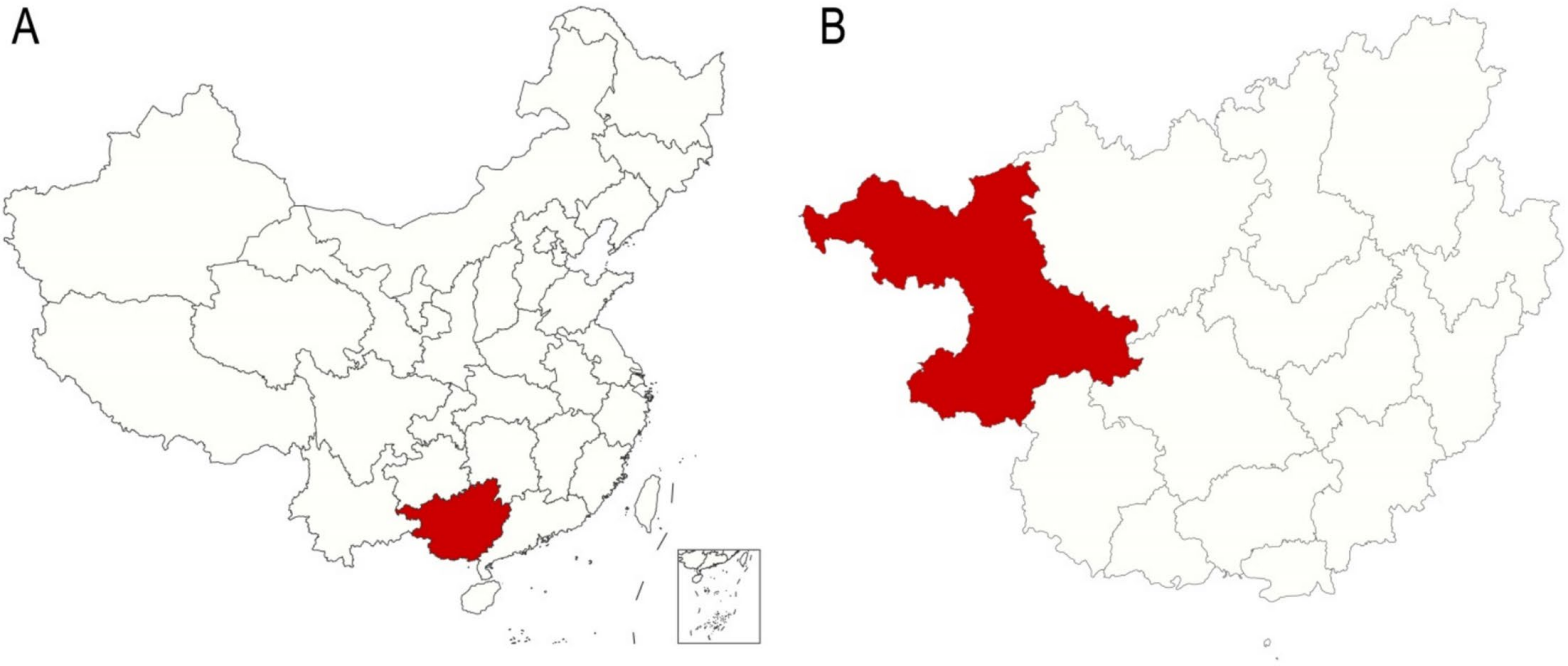
Localization of the underdeveloped minority areas in Guangxi, China. **(A)** A map of China with the Guangxi Zhuang Autonomous Region marked in red. **(B)** A map of the Guangxi Zhuang Autonomous Region with the underdeveloped minority areas marked in red.

The underdeveloped minority areas in China, covering a total area of 36,300 km^2^ (Figure 1). This region is home to various ethnic groups, including Han, Man, Zhuang, Yao, Miao, Yi, Buyi and Gelao, with a total population exceeding 3.5 million (13).

### Sample size

The sample size used was calculated based on the formula for the sampling survey (14):


$$n=\frac{\mu_{\frac{\alpha }{2}}^{2}\pi (1-\pi )}{\delta^{2}}$$

where π is the overall rate. If π had several reference values at the same time, the value should be closest to 0.5. If blinded to the population, π was set to 0.5. Because in this study, π(1-π) = 0.5^2^ = 0.25 which was the maximum value, therefore n could not be too small. Tolerance error ***δ*** = 0.05, *α* = 0.05, μ_α/2_ = 1.96, then n = 385.

### Statistical analysis

The maps of China and the Guangxi Zhuang Autonomous Region were plotted using R language. The data were visualized using WPS Office software (China) (15), which was also used to organize and categorize the data, and calculate the descriptive statistics [e.g., medians, standard deviations, averages and interquartile ranges (IQR)].

SPSS version 26.0 software (IBM Corporation, United States) was used for statistical analyses. Data were tested for normality using the Shapiro–Wilk test. Normally distributed data were presented as means±SDs, whereas data with non-normal distribution were presented as medians and IQRs. The chi-squared, Mann–Whitney U and Kruskal–Wallis tests were performed to detect differences between groups for non-normally distributed categorical and quantitative variables. *Post-hoc* comparisons between two groups were conducted using the chi-squared with the Z tests. The Monte Carlo method was applied to the chi-squared test when the data were composed of less than 5 cases or when the total sample sizes were fewer than 40 in analysis of the etiology of burn cases.

To satisfy the assumptions of multiple linear regression, particularly the normality of residuals, log-transformation of the dependent variables (length of hospital stay (LOHS) and cost) was performed. This decision was based on the observation that the standardized residuals of the original models deviated from normality, as assessed by diagnostic plots and normality tests. The log-transformation is a commonly used approach to stabilize variance and improve the normality of model residuals in skewed data. Variables were selected for multivariate analyses based on clinical relevance and significance in univariate comparisons (*p* < 0.10). Standardized beta coefficients (SBC) were reported to assess the relative importance of predictors in linear regression. Model assumptions, including normality and linearity, were verified before regression modeling. To improve comparability and interpretability, continuous variables with skewed distributions were log-transformed where appropriate. All analyses were two-tailed, and *p* < 0.05 was considered statistically significant. And all eligible cases admitted between January 2014 and December 2020 were included. The number of events per variable in each model was sufficient to ensure the reliability of the results.

## Results

### Sociodemographic characteristics

**Figure 2:**
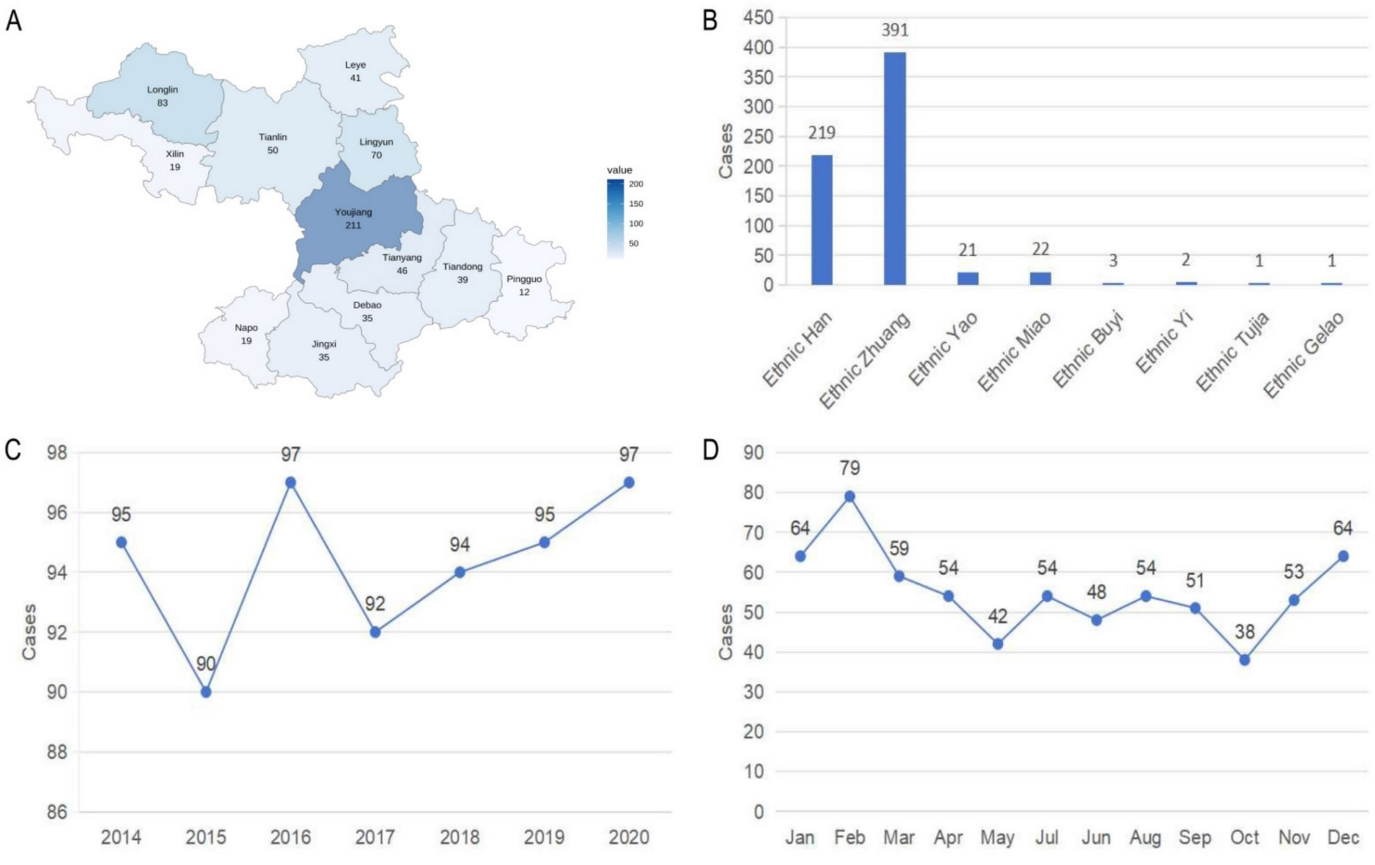
Distribution of sociodemographic characteristics. **(A)** Distribution of 660 pediatric burns in underdeveloped minority areas. **(B)** Distribution of 660 pediatric burns as depicted by ethnicity. **(C)** Distribution of 660 pediatric burns as depicted by year. **(D)** Distribution of 660 pediatric burns as depicted by month.

The 698 children from underdeveloped minority areas were hospitalized due to burns from January 2014 to December 2020. However, 38 burn cases were excluded due to missing medical information. The 38 excluded cases included 10, 14, 5, 4, 3, 1 and 1 cases in 2014 to 2020, respectively. Among them, there were 14, 1, 1, 2 and 20 from Han, Man, Miao, Yao and Zhuang ethnic nationalities, respectively. Figure 2 presents the sociodemographic distribution of the 660 pediatric burn patients from 12 different counties in the underdeveloped minority areas. The 660 burn patients represented 8 different ethnic groups. The Zhuang ethnic group had the highest number of cases, with 391 children, followed by the Han ethnic group, with 219 children. Other minority groups collectively contributed 50 children. The distribution of these 660 pediatric burn patients spans from 2014 to 2020, with annual counts ranging from 90 to 97. The years 2016 and 2020 each had the highest number of cases at 97, while 2015 had the fewest, at 90. The monthly distribution analysis revealed that February had the highest number of cases, with 79 children, followed by December and January, each with 64 cases. October and May had the fewest cases, with 38 and 42 children, respectively.

### Distribution of associated factors

**Figure 3:**
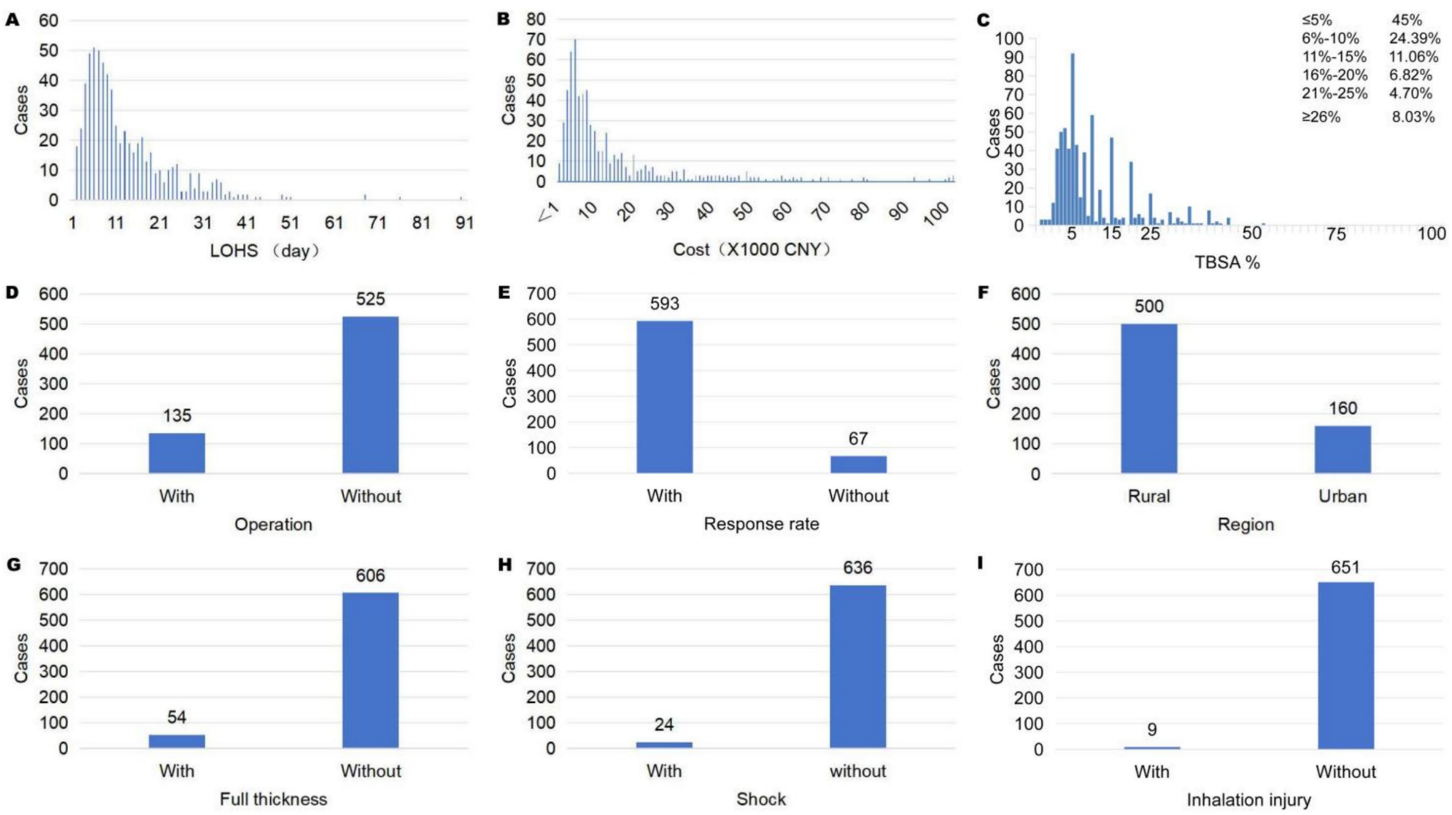
**(A)** Distribution of LOHS. **(B)** Distribution of cost. **(C)** Distribution of TBSA%. **(D)** Distribution of operation. **(E)** Distribution of response rate. **(F)** Distribution of region. **(G)** Distribution of full thickness. **(H)** Distribution of shock. **(I)** Distribution of inhalation injury.

The distribution of relevant variables among the 660 pediatric burn patients is shown in Figure 3. The length of hospital stay (LOHS) ranged from 1 to 89 days, with 381 cases having LOHS ≤10 days (Figure 3A). Hospitalization cost (COST) ranged from 119.65 CNY to 154,144.07 CNY, and 415 patients had total costs within 10,000 CNY (Figure 3B). As for burn severity, 45.00% of the patients sustained burns covering 0–5% of the total body surface area (TBSA), 24.39% had 6–10% TBSA, 11.06% had 11–15% TBSA, and 19.55% had burns involving more than 15% TBSA (Figure 3C). A total of 135 children underwent surgical intervention as required by clinical condition (Figure 3D). Among the cohort, 593 patients were classified into the response rate group based on caregivers’ acceptance of standardized treatment (Figure 3E). A total of 500 children were from rural areas (Figure 3F). Full-thickness burns were present in 54 cases (Figure 3G), 24 children experienced burn shock (Figure 3H), and 9 children had concomitant inhalation injury (Figure 3I).

### Etiology and distribution

**Figure 4:**
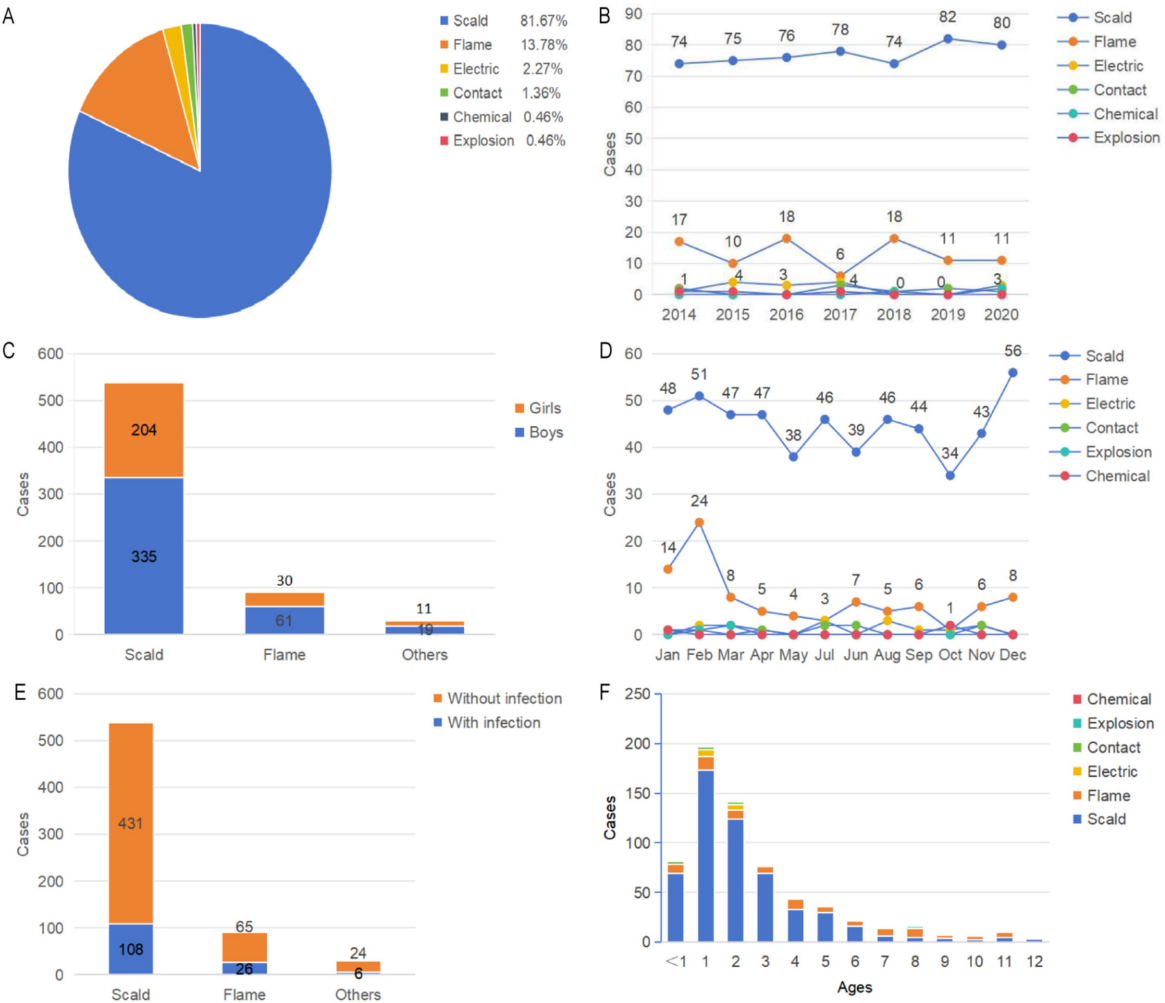
Distribution of the etiology and severity of burn cases. **(A)** The proportion of different causes of burns. **(B)** Distribution of etiology as depicted by year. **(C)** Distribution of gender as depicted by the etiology of burn cases. **(D)** Distribution of etiology as depicted by month. **(E)** Distribution of burn infections as depicted by etiology of burn cases. **(F)** Distribution of etiology as depicted by age.

In this study of pediatric burn patients from underdeveloped minority areas, the causes of burns among the 660 children were predominantly scalds (81.67%), followed by flame burns (13.78%), electrical burns (2.27%), contact burns (1.36%), chemical burns (0.46%), and explosions (0.46%). Scalds, followed by flame burns, formed the majority of the cases with only 30 resulting from other causes (Figures 4A–C). The distribution of scalds showed no significant variation across the period of study. The highest number of flame burns occurred in 2016 and 2018, with 18 cases each, while the lowest number was in 2017, with 6 cases (Figure 4B). Both scalds and flame burns peaked in winter months (December to February), with the highest number of flame burns in February (24 cases) and the highest number of scalds in December (56 cases). Burns from other causes peaked in June. The monthly distribution of burn etiologies differed significantly (*p* = 0.003). The etiology distribution in February and June differed significantly when compared to other months (*p* < 0.05; Figure 4D; Table 1). Of the 140 children with burn infections, 108 cases were scald group, 26 cases were flame group and 6 cases were others group. There was no significant difference in the distribution of etiologies (*p* < 0.05; Figure 4E; Table 1).

**Table 1:** Analysis of the etiology of burn cases.

	Etiology			*p*-value
	Scald	Flame	Electric/Contact/Chemical/Explosion	
Age				
<1	69	9	3	*p*<0.001
1–3	366	30	20	
4–6	79	21	3	
7–12	25	31	4	
Gender				
Boys	335	61	19	*p* = 0.671
Girls	204	30	11	
Months				
Jan	48	14	2	*p*<0.003
Feb	51	24	4	
Mar	47	8	4	
Apr	47	5	2	
May	38	4	0	
Jun	46	3	5	
Jul	39	7	2	
Aug	46	5	3	
Sep	44	6	1	
Oct	34	1	3	
Nov	43	6	4	
Dec	56	8	0	
Region				
Rural	401	77	22	*p* = 0.104
Urban	138	14	8	
Ethnics				
Minority	353	64	24	*p* = 0.193
Han	186	27	6	
Burn infection				
With	108	26	6	*p* = 0.181
Without	431	65	24	

Scalds and flame burns were more common in boys, while cases of other causes differ little (Figure 4C). The peak incidence of burns for all causes occurred in children aged 1–3 years (Figure 4F). More than half of the scalds occurred in this age group, with 366 out of 660 cases (55.45%). The incidence of flame burns in children aged 1–3 years was 30 out of 91 cases (32.97%), while burns from other causes in this age group accounted for 20 cases (66.67%). In the 7–12 years age group, the proportion of flame burns was the highest, with 31 cases. The prevalence of burns from various causes was higher among rural children and children from minority ethnic groups compared to urban and Han children (Table 1).

### Predictive factors of LOHS

The median LOHS was 9 days (IQR: 5, 16). Table 2 shows the multiple linear regression of factors associated with LOHS in cases where P was less than 0.05. Among all the factors, a higher response rate had the greatest impact on prolonging LOHS (standardized beta coefficient (SBC) = 0.391, *p* < 0.001), followed by larger a TBSA (SBC = 0.357, *p* < 0.001) and the presence of an operation (SBC = 0.350, *p* < 0.001). However, burn infections significantly increased the LOHS (SBC = 0.085, *p* = 0.003). Compared to scalding, the etiology categorized as “others” had a negative SBC of −0.059 (*p* = 0.033).

**Table 2:** Multiple linear regression analysis of the factors associated with LOHS.

	Unstandardized beta coefficients		Standardized beta coefficients	*t*	*p* value	VIF
	Beta	SE				
Response rate	1.084	0.076	0.391	14.319	<0.001	1.109
TBSA	0.031	0.003	0.357	10.784	<0.001	1.630
Operation	0.726	0.063	0.350	11.565	<0.001	1.360
Burn infection	0.174	0.059	0.085	2.939	0.003	1.240
Etiology Others	−0.238	0.111	−0.059	−2.142	0.033	1.136

The natural logarithm (ln) of LOHS was used for the multivariate regression analysis to meet the normality assumption. Dependent variable: Ln (LOHS), Enter Method, Constant = 0.640, R Square = 0.565, adjusted R Square = 0.557, Durbin-Watson = 1.800, F = 64.650, *p*<0.001. Etiologies were categorized as scalds, flame and others. Others: contact/electric/chemical/explosion in etiology. The factors included in the regression model were burn infections, TBSA, inhalation injuries, full-thickness burns, burn shock, operation, region, age, gender, response rate, ethnicity and etiology.

### Predictive factors of cost

The median cost of pediatric burn cases was 7,558 CNY (IQR: 4217, 16,633). Table 3 shows the multiple linear regression of factors associated with the costs in cases where P was less than 0.05. Among all the factors, a higher LOHS had the greatest impact on increasing the cost (SBC = 0.468, *p* < 0.001), followed by a larger TBSA (SBC = 0.306, *p* < 0.001), the presence of an operation (SBC = 0.215, *p* < 0.001) and the response rate (SBC = 0.120, *p* < 0.001). Compared to urban areas, the region categorized as rural had a SBC of 0.081 (*p* < 0.001), and compared to scalding, the etiology categorized as “others” had a negative SBC of −0.073 (*p* < 0.001).

**Table 3:** Multiple linear regression analysis of the factors associated with cost.

	Unstandardized beta coefficients		Standardized beta coefficients	*t*	*p* value	VIF
	Beta	SE				
LOHS	0.046	0.003	0.468	16.075	<0.001	2.385
TBSA	0.033	0.003	0.306	11.590	<0.001	1.965
Operation	0.555	0.064	0.215	8.620	<0.001	1.748
Response rate	0.412	0.072	0.120	5.693	<0.001	1.240
Region	0.197	0.047	0.081	4.162	<0.001	1.066
Etiology Others	−0.366	0.101	−0.073	−3.643	<0.001	1.138

The natural logarithm (ln) of cost was used for the multivariate regression analysis in order to meet the normality assumption. Dependent variable: Ln (cost), Enter Method, Constant = 7.313, R Square = 0.770, adjusted R Square = 0.765, Durbin-Watson = 1.780, F = 154.619, *p*<0.001. Etiologies were categorized as scalds, flame and others. Others: contact/electric/chemical/explosion in etiology. The factors included in the regression model were burn infection, TBSA, inhalation injury, full-thickness burns, burn shock, operation, region, age, gender, response rate, ethnicity, etiology and LOHS.

## Discussion

Unintentional injuries among children encompass a wide range of incidents, including traffic injuries, drowning, poisoning, burns and falls (16). Among these, burns are particularly common and can result in severe physical and psychological damage (17). Pediatric burn patients constitute a significant proportion of hospitalized patients globally (7). This study primarily analyzed the epidemiology and burden of disease associated factors for pediatric burns in underdeveloped minority areas of Guangxi, China over a recent seven period. It initially explores the epidemiological characteristics of burns in these areas and proposes corresponding prevention strategies based on the factors related to burns, providing insights for underdeveloped and poorer regions worldwide.

The study sample included 660 cases from 12 different counties in underdeveloped minority areas, encompassing seven different ethnic groups. Specifically, the sample comprised of 391, 219 and 50 children from the Zhuang, Han and other minority ethnic groups, respectively. The ethnic composition of these areas differs significantly from the national average in China, where the Han ethnic group constitutes over 90% of the population. In contrast, in Baise, a prefecture-level city within the Guangxi Zhuang Autonomous Region, the Han population accounts for only 16.47% of the total, with the Zhuang and other minority populations making up 83.53% (18). This ethnic distribution was reflected in the pediatric burn cases in our study, where the pediatric burns in the minority groups accounted for 66.8% (441 out of 660 cases) of the total. These findings underscore the importance of recognizing regional and ethnic disparities in burn epidemiology, which are often masked in national-level statistics.

The high proportion of burn injuries among minority children may be partly attributed to socioeconomic and cultural factors prevalent in these regions. Many minority families reside in remote rural areas with limited access to healthcare resources, low income levels, and lower health literacy, all of which can increase the risk of both burn injury and suboptimal treatment adherence (19). Furthermore, traditional child-rearing practices—such as multi-generational caregiving and limited supervision—combined with environmental hazards (e.g., flames, boiling water, and poor housing infrastructure), may contribute significantly to the elevated risk (20). Additionally, cultural beliefs and reliance on traditional remedies can influence treatment-seeking behavior (21). In our study, a subset of caregivers voluntarily withdrew their children from hospital care in favor of non-standard or folk treatment methods, resulting in a lower response rate to standardized care. This highlights the need for culturally sensitive health education programs and community-based interventions tailored to the specific needs of ethnic minority populations. Overall, these findings suggest that burn prevention and treatment strategies must be adapted to the unique cultural, economic, and geographic contexts of underdeveloped minority regions in order to effectively reduce burn-related morbidity in children.

Given the unique sociocultural and economic context of underdeveloped ethnic minority regions, targeted and culturally sensitive burn prevention strategies are urgently needed. Public health education efforts should be tailored to the local linguistic and cultural landscape, utilizing bilingual materials and involving trusted community leaders to disseminate burn prevention messages (22). In rural areas, where open flames and hot liquids are common in daily life, household-level interventions—such as promoting safe cooking practices and distributing low-cost safety equipment—can be effective in reducing burn risks among children (23).

Strengthening primary healthcare systems is also essential. This includes training rural healthcare workers in the early recognition and management of burns and improving referral networks to ensure timely access to specialized care (24). At the same time, traditional beliefs and treatment preferences must be acknowledged and respectfully addressed to enhance guardians’ acceptance of standardized medical treatment. Engaging with cultural norms rather than attempting to override them can foster trust and improve treatment adherence (25).

In addition to health systems, social systems, such as schools, communities, families, and digital platforms, can be used to promote health. Academic institutions and non-governmental organizations should also be encouraged to support evidence-based program development, monitoring, and evaluation (26). Multisectoral collaboration—across health, education, maternal and child services, and local governance—can facilitate the integration of burn prevention into broader child health and rural development agendas (27). Moreover, policy-level support is necessary to ensure the sustainability of these interventions (28). Collectively, these strategies can help reduce pediatric burn incidence and morbidity in vulnerable ethnic minority populations and contribute to health equity in burn care across China’s diverse regions.

Consistent with previous studies (29–31), the ratio of boys to girls among the 660 burn children in this study was 1.69:1, indicating that boys were the primary gender affected by burns. Boys are more prone to burns from various causes but they also suffer more severe burns. This may be because boys are naturally more inclined to engage in riskier behaviors, have a stronger sense of curiosity and a greater desire to explore new environments. This could also be related to the different associated factors that girls are exposed to (32). For example, a mother and be involved with cooking making tea and ironing, while boys may be involved in lighting fireworks and so on.

The proportion of burn cases among children varied significantly with age. Globally, a large portion of pediatric burn cases occurs in children under the age of five. In Europe, this age group accounts for 50–80% of all pediatric burns (33). In a study conducted by Han et al. (2022), pediatric burns were classified into four groups based on age-related physiological characteristics: infants (≤1 year), toddlers (>1 year and ≤3 years), preschool children (>3 years and ≤7 years) and school-age children (>7 years and ≤14 years). The study results indicated that burns were most common in patients aged 1–3 years. Additionally, the findings showed that although children under the age of three had a high incidence of burns, the severity of burns in this age group was relatively lower when compared to children over the age of three. This trend can be attributed to the developmental and behavioral characteristics of children within this age range (34).

In a systematic review by Veldman et al. on the association between physical activity and indicators of health and development in children under the age of five, it was clear that in 3–5 year olds, physical activity and motor skills were positively correlated with their cognitive abilities. However, there is still a lack of in depth studies on the relationships between physical activity and health indicators and developmental outcomes (such as motor development, cognitive development and social–emotional development) in children under the age of three (35). It is likely because the physical activity and motor skills of children aged 1–3 years are continuously developing, but their cognitive abilities do not increase at the same pace, these reasons could lead to a higher likelihood of exposure to dangers. As a result, children in the 1–3 year age group have a higher prevalence of burns as well as other accidents.

In this study, scalds were the most common cause of burns, accounting for 81.67% of all cases and flames were the second most common cause (13.78%). Other rarer causes of burns included electrical, contact, chemical and explosion injuries, similar to the findings in other studies (7, 36). Physiologically, burns in young children may be more severe because their skin layers and insulating subcutaneous tissues are thin when compared to those of older children and adults. Burns in children can also result in a faster and a higher percentage of fluid loss than in adults, necessitating relatively larger fluid resuscitation (37).

The distribution of burn types observed in this study—predominantly scalds, followed by flame burns, with relatively few electrical, chemical, or other types—reflects the unique socioeconomic and environmental characteristics of underdeveloped ethnic minority regions. Scald injuries, particularly those caused by hot water, were the most common, which can be attributed to traditional domestic practices. In many rural households, boiling water is frequently used for bathing, cooking, and drinking due to the lack of centralized hot water systems. These households often rely on open flames or simple stoves for heating water, and hot liquids are commonly left in open containers within the reach of young children. Additionally, child supervision is often limited, particularly in multigenerational households where older adult caregivers may lack sufficient awareness of burn risks (38, 39). Flame burns were the second most common type, likely due to the continued reliance on open firewood or coal stoves for cooking and heating in these communities. Poor ventilation, overcrowded living spaces, and the absence of child safety barriers further increase the risk of fire-related injuries (40). In contrast, electrical and chemical burns were rare. This may be due to the lower levels of industrialization, limited access to electrical appliances, and minimal use of chemical cleaning agents or industrial materials in daily life. These patterns highlight the importance of region-specific burn prevention strategies that focus on improving household safety, promoting awareness of scald and fire hazards, and enhancing caregiver supervision in rural minority communities.

Other preventive measures for pediatric burns should be community-based and multi-strategic. These measures include the installment of smoke alarms, adjusting household tap water temperatures and setting safe preset temperatures on water heaters, using flame-retardant materials for children’s clothing, improving fire sprinkler systems, ensuring electrical safety, legislating and educating about fireworks, placing hot drinks/food out of their reach and storing matches and lighters in inaccessible places (16). The risk of accidental injury in children is influenced by three main factors: (1) a child’s physical and cognitive abilities, which is primarily determined by age, (2) environmental and neighborhood factors and (3) social factors, such as inadequate care and insufficient adult supervision (41). Therefore, targeted preventive measures and health education are essential prerequisite strategies that must be implemented in order to reduce the incidence of pediatric burns. It is important to note that pediatric burn victims in underdeveloped ethnic minority regions often face compounded challenges, including financial hardship, inadequate infrastructure, and limited access to health and educational resources (38, 39). Therefore, burn prevention strategies in these settings must emphasize practicality, cultural sensitivity, and affordability. Effective measures may include promoting safe hot water handling practices, constructing simple physical barriers around open flames and cooking stoves, and implementing community education programs using bilingual, pictorial materials tailored to local literacy levels (40). Additionally, existing rural healthcare resources—such as village clinics and community health workers—can be leveraged to deliver targeted home safety interventions. Low-cost tools such as elevated storage for hazardous items, visual safety cues within the household, and village-led safe-kitchen campaigns may serve as scalable and sustainable models to mitigate the risk of pediatric burns in resource-constrained environments (42).

This study revealed a distinct seasonal distribution of pediatric burn etiologies: scalds were most common in winter, flame burns peaked in February, and other causes of burns were more frequent in June. These findings are consistent with prior research suggesting that lifestyle, climatic conditions, and cultural festivities significantly influence burn incidence in different geographic settings (39).

In underdeveloped ethnic minority areas, such seasonal trends are further intensified by specific environmental, behavioral, and caregiving factors. During winter, the absence of central heating systems leads many families to rely on open-flame stoves, hot water basins, and charcoal braziers for warmth and hygiene purposes. These traditional heating and cooking methods, often used in crowded and poorly ventilated spaces, substantially increase the exposure risk of young children to thermal hazards (40).

Moreover, young children are frequently cared for by older adult relatives in multi-generational households. These caregivers may be less capable of providing continuous supervision, particularly when multitasking with household chores. At the same time, toddlers’ natural curiosity and limited understanding of environmental risks make them highly vulnerable to burn injuries (17). The winter season also necessitates the use of layered or bulky clothing, which may hinder prompt removal in the event of a burn and potentially increase burn depth (43).

In certain minority communities, it is customary to place hot water containers on the ground or to allow children unrestricted access to cooking areas. This practice, coupled with the absence of physical safety barriers, heightens the likelihood of scalds from accidental spills (44). In addition, national holidays such as the Spring Festival often involve widespread use of fireworks and firecrackers, contributing to the spike in flame-related injuries observed in January and February (4, 45). Based on these findings, targeted preventive strategies should be implemented including the use of water at temperatures below 40°C when bathing, setting the temperature of water heaters to below 50°C and enhanced supervision of children as water temperatures of 65–70°C can cause severe scalds (34).

Taken together, these findings underscore the importance of implementing seasonally tailored prevention strategies. Public health interventions—such as winter-focused burn awareness campaigns, low-cost modifications to household fire and water use practices, and culturally sensitive caregiver education—are critical for reducing the pediatric burn burden in resource-limited ethnic minority areas.

It is well known that the burden of disease related to pediatric burns, including LOHS and cost, is considerable (34, 46). In this study, the median LOHS was 9 days (IQR: 5–16) and the median cost was 7,558 CNY (IQR: 4217–16,633 CNY). The LOHS and costs were lower than those reported in studies from other regions in China (7). In this study, the median TBSA was 6% (IQR: 4–15%). Total TBSA represents the percentage of the body’s surface area affected by burns (47). In this study, the proportion of pediatric burns with TBSA <5% was 45%, and the proportion with TBSA <10% was nearly 70%. Lower TBSA was significantly associated with both shorter length of hospital stay (LOHS) and reduced hospitalization cost. This correlation can be attributed to the fact that limited burn surface area typically reflects a lower severity of injury, which in turn requires less intensive medical intervention. Children with small-area burns are more likely to recover without the need for surgical procedures, prolonged wound management, or intensive care, all of which are major contributors to both extended hospitalization and higher treatment costs (48). Furthermore, smaller TBSA injuries carry a lower risk of complications such as infection, shock, or inhalation injury, which often necessitate additional diagnostic testing, intravenous antibiotics, or respiratory support. As a result, patients with minor burns tend to follow a more straightforward clinical course and are discharged more quickly, leading to significantly reduced resource utilization.

These findings are consistent with previous studies demonstrating that TBSA is a primary determinant of both clinical severity and economic burden in pediatric burn patients (49, 50). Thus, TBSA can serve not only as a prognostic indicator but also as a practical predictor of healthcare resource consumption.

Demographic variables, the severity of burn injuries and burn treatment methods can all potentially be prognostic factors affecting the LOHS in burn patients (51). The variables affecting the LOHS for pediatric burn patients in underdeveloped minority areas of Guangxi, China, include response rate, TBSA, surgery, burn infection and other etiologies. The response rate was defined as the proportion of pediatric burn patients whose caregivers, after medical consultation and explanation by clinical staff, agreed to adhere to standardized burn treatment protocols throughout hospitalization. In contrast, cases in which caregivers voluntarily discharged the child from the hospital—often due to financial constraints or preference for non-standard or traditional treatment approaches—were categorized as non-response.

A higher response rate was significantly associated with increased length of hospital stay (LOHS) and higher hospitalization costs. This correlation can be attributed to the fact that patients who remain in care for the full course of standardized burn treatment receive more comprehensive clinical management, including advanced wound care, surgical interventions when necessary, and monitoring for complications. These interventions, while improving clinical outcomes, inevitably require prolonged hospital stays and greater resource utilization, resulting in elevated costs.

In contrast, non-response cases often represent premature discharge or treatment abandonment due to financial hardship or reliance on folk remedies. Such cases typically forgo critical aspects of standardized care, resulting in reduced hospital stays and lower immediate costs—though at the potential expense of long-term health outcomes. Therefore, while a higher response rate reflects improved treatment adherence and care continuity, it also corresponds with increased medical expenditures and utilization of hospital resources. These findings underscore the dual challenge in underdeveloped minority regions: improving access and adherence to effective care, while also ensuring affordability and health system sustainability (38).

The classification of burn wounds based on severity or the ABA system indicated that pediatric burns with <10% TBSA or <2% full-thickness TBSA were often treated on an outpatient basis (52). However, in this study, many caregivers, out of concern for their children, insisted on hospitalization even for small-area burns. In this study, a notable number of pediatric patients with minor burn injuries were hospitalized and completed the full course of standardized treatment. This pattern may initially appear to contradict global burn management guidelines, which typically recommend outpatient care for small TBSA burns. However, such decisions must be contextualized within the unique socioeconomic and health system realities of underdeveloped minority regions. Caregivers in these areas often perceive hospitalization not only as a treatment necessity, but also as a safety net—particularly in light of limited access to outpatient follow-up, poor transportation infrastructure, and inadequate community health services. Given the limited health literacy and lack of home-based wound care knowledge, hospital admission—even for minor burns—ensures safe monitoring, appropriate wound management, and timely identification of complications, such as infection or dehydration. Furthermore, the scarcity of trained healthcare workers and burn care expertise in rural clinics makes hospital-based care the only reliable option. Therefore, these seemingly “prolonged” hospital stays should be interpreted not as inefficient resource use, but rather as a rational adaptation to structural healthcare deficiencies. This also underscores the need to reframe international clinical guidelines through a health equity lens, adapting them to the realities of low-resource, underserved settings.

Surgical intervention emerged as a significant factor associated with increased length of hospital stay (LOHS) and total medical costs in our multivariate regression analysis. This is consistent with clinical expectations, as burn surgery—such as debridement, skin grafting, or wound reconstruction—requires perioperative care, extended wound monitoring, and postoperative management, all of which contribute to prolonged hospitalization and increased resource utilization (53). In the context of underdeveloped ethnic minority regions, the impact of surgery on LOHS and cost may be even more pronounced. First, due to limited healthcare infrastructure, perioperative efficiency is often lower, and recovery monitoring may be more cautious and prolonged to avoid complications in resource-scarce settings (38). Second, surgical decisions may be influenced not only by clinical severity but also by caregiver expectations and healthcare providers’ efforts to optimize long-term outcomes within one hospitalization episode, given the difficulty of follow-up care in remote areas (54). Furthermore, surgical needs often reflect deeper or more extensive burns, which are inherently associated with more severe physiological stress, infection risk, and psychosocial burden—factors that further drive up LOHS and cost (43, 49). Therefore, surgery should be interpreted not merely as a procedural event, but as a proxy indicator of injury severity, resource intensity, and systemic constraints in these regions. These findings emphasize the need to improve access to safe, timely, and cost-effective surgical care, while also investing in preventive strategies that reduce the incidence of burns requiring surgery in the first place.

Although the standardized coefficient (SCB = 0.081, *p* < 0.001) for the variable region in the cost model appears relatively small, its real-world significance is considerable when contextualized within the epidemiology of pediatric burns in underdeveloped ethnic minority areas. In our cohort, 75.8% (500 out of 660) of burn cases were from rural regions. These areas are typically characterized by delayed access to emergency care, inadequate first aid knowledge, insufficient burn treatment infrastructure, and limited postoperative rehabilitation services, all of which may elevate the financial burden associated with hospitalization and recovery—even for less severe burns. Importantly, in rural households where economic resources are scarce, even modest increases in cost represent a disproportionately high financial burden. Moreover, cultural beliefs and low health literacy in rural ethnic communities may contribute to treatment non-adherence or extended inpatient stays for conditions that could otherwise be managed in outpatient settings. Therefore, the region variable should not merely be interpreted as a geographic indicator, but as a surrogate marker for deeper structural and health system inequities that significantly influence burn care outcomes in marginalized populations. These findings highlight the urgent need for targeted burn prevention and care delivery strategies in rural minority settings, including community-based first aid training, mobile wound care units, subsidized treatment plans, and rural-focused policy interventions (38, 39, 55).

This study strictly categorized burn infections using the ABA Burn Infection Classification method. The ABA’s diagnostic criteria for burn sepsis is a diagnostic system established based on infection-related systemic responses, organ dysfunction, and microbial infection diagnostic indicators. Its significance lies in enhancing the specificity of burn sepsis diagnosis and avoiding false positives due to over-reliance on the SIRS diagnostic criteria. Early diagnosis of burn sepsis helps guide antibiotic use, fluid resuscitation, and control of the source of infection, thereby reducing the incidence of severe complications after burn infections. Although burn infection was found to be a statistically significant predictor of length of hospital stay (LOHS) in our multivariate linear regression model (*p* = 0.003), the standardized coefficient (SCB = 0.085) was relatively small, indicating a limited strength of association. This suggests that while infection does contribute to prolonged hospitalization, its independent impact—when controlling for other variables—may be modest. From a clinical perspective, this can be interpreted in several ways. First, the early identification and management of burn infections in the study setting may have mitigated their influence on hospitalization duration. Second, other factors—such as burn depth, surgical interventions, and response rate—may exert a stronger influence on LOHS and potentially attenuate the relative effect of infection in the model. Therefore, while statistically significant, the small SCB suggests that burn infection should be considered a contributing—but not dominant—factor in determining LOHS within this population. This highlights the importance of interpreting statistical results in the context of local clinical practices and resource environments.

In both the LOHS and cost regression models, burns classified under the “others” etiology category emerged as statistically significant protective factors when compared to scald injuries (*p* < 0.05). However, the standardized coefficients (SCBs) for this variable were consistently small (all < 0.1), suggesting a minimal independent effect size. This finding indicates that although the presence of “other” types of burns (e.g., electrical, chemical, contact) is associated with slightly lower hospitalization durations and treatment costs than scalds, the actual magnitude of this effect is limited. From a practical standpoint, this may be attributed to the relatively small number of “others” cases in the dataset and the variability within this heterogeneous category, which encompasses diverse etiologies with differing severities and care needs. Moreover, in the context of underdeveloped regions, scalds are more common and often involve very young children, leading to longer hospital stays due to parental concern, infection monitoring, or precautionary treatment. By contrast, “other” burn types may be more localized or smaller TBSA, resulting in a statistical trend toward shorter LOHS and lower costs, albeit with limited real-world impact. Overall, while statistically significant, the protective role of “other” etiologies should be interpreted with caution due to the small effect sizes and the broad heterogeneity within this category.

Burn shock, as an acute circulatory failure, typically occurs in the early stages of burns (within 48 h after injury). Standardized fluid resuscitation therapy driven by clinical guidelines may significantly shorten the duration of shock, making its cumulative effect on the hospital stay less impactful compared to persistent factors (such as burn infection, surgery, and TBSA). Additionally, 24 out of 660 (3.6%) experienced burn shock, which is a relatively low proportion among children with burns. This low proportion is reflected in both the annual comparisons and the value contribution to the burden model of burn-injured children, thus failing to demonstrate significant impact in the study.

The above discussion is based on the results of this study, however in the prevention of burns in children. Passive pediatric burn prevention strategies aim to prevent burns by modifying the child’s surrounding environment, while active ones aim to achieve this by changing the child’s attitude and behavior. Both strategies can effectively reduce the incidence of pediatric burns although the former may be more effective, particularly for participants with lower compliance (56). To strengthen pediatric burn prevention strategies, it is critical to explicitly recognize adult caregivers—particularly young parents aged 20 to 35 years—as parallel targets for intervention. This demographic group aligns with the age range of most caregivers in our study population and plays a central role in shaping the daily safety environments of children. Given that young children are heavily reliant on adults for protection from environmental hazards, caregiver behavior, awareness, and supervision practices are among the most influential modifiable associated factors in burn prevention. This view has also been confirmed in the previous research (18, 57).

Educational and behavioral interventions aimed at this group can have substantial downstream impact. Tailored programs—delivered through community health workers, village clinics, or school-based parent outreach—could focus on basic burn hazard identification, first-aid skills, safe cooking and bathing practices, and the importance of child supervision in high-risk contexts. In regions with low literacy, the use of culturally appropriate visual aids and bilingual materials may enhance comprehension and retention (58, 59).

Targeting caregivers in this age group also presents an opportunity for intergenerational impact: young parents who adopt safer practices are more likely to model and transmit these behaviors to future generations. Integrating caregiver-focused education into broader child health promotion platforms—such as maternal and child health services or rural public health campaigns—may therefore represent a highly feasible and cost-effective strategy for reducing pediatric burn incidence in underdeveloped regions (60, 61). Additionally, cooperation among the government officials, healthcare providers, families, teachers and the society as a whole is necessary to reduce the prevalence, mortality and disease burden of pediatric burns worldwide (62). Totally, The burden of pediatric burns are associated with multiple factors, these results will provide the basis and support for assessing the epidemiology and serious consequences of pediatric burns in this region.

Burn prevention remains one of the most cost-effective strategies for reducing the incidence and burden of pediatric burn injuries (42). Evidence from global injury prevention programs suggests that interventions aiming to shift individual behaviors and attitudes—as well as to enhance the safety of physical environments—can significantly lower injury risk (63). In underdeveloped minority regions, where children are exposed to multiple burn risk factors including unsafe housing, limited supervision, and cultural reliance on traditional practices, prevention efforts must adopt a multifaceted approach. Combining active strategies—such as health education, caregiver training, and community engagement—with passive strategies—like infrastructure improvement, safe kitchen design, and flame barriers—is likely to yield the most sustainable impact. Tailoring these interventions to the socioeconomic, cultural, and linguistic context of minority populations is critical. This study highlights the urgent need for integrated, community-based, and culturally sensitive burn prevention models to effectively protect vulnerable children in resource-limited settings.

The data show that thermal injuries account for 81.67% of all causes of childhood burns. Due to their lower thermodynamic characteristics, the incidence of full-thickness burns is significantly lower than that of other types of burns such as flame or electric shock injuries. In the study, only 8.18% of children had combined full-thickness burns. When constructing the multivariate regression model, the regression coefficient of the full-thickness burn variable did not reach the statistical significance threshold and was excluded from the model. It is recommended that future studies adopt a prospective cohort design, using stratified sampling strategies or expanding inclusion criteria to further precisely quantify the contribution of burn depth dimensions in predictive models.

We conducted an epidemiological study on the disease burden of pediatric burns in underdeveloped minority areas but there were some limitations. Our sample of 660 pediatric burn cases was collected from only two hospitals. Additionally, aside from the larger numbers of Han and Zhuang ethnic children included, the number of children from other minority groups was limited, making our results not fully representative of minorities other than the Zhuang. Since scalds and flames often cause pediatric burns, the number of cases from other causes was relatively small. Therefore, the representativeness of the etiological analysis may vary. More comprehensive studies are needed to further explore the epidemiological patterns and associated factors of the disease burden for pediatric burns in underdeveloped minority areas. It is well known that retrospective studies can be affected by incomplete or inaccurate data, impacting the reliability of the research results. Since the selection of subjects is based when using historical data, selection bias may affect the representativeness of our findings. Retrospective studies cannot always establish temporal sequence and causality, thereby limiting causal inferences. Controlling for all possible confounding variables can be challenging and this can add complexity to the study results. These are some of the limitations in this study. To address these issues, we need to design prospective studies to collect future data. Additionally, conducting multi-center collaborative research will increase the sample size and data diversity, thus improving the external validity of the results obtained. These approaches are the basis of our future research plans.

## Conclusion

This study describes the epidemiological characteristics of pediatric burns in underdeveloped minority areas of Guangxi, China, from 2014 to 2020, and analyzes the associated factors for length of hospital stay (LOHS), and total cost. We found that children under the age of three, particularly those sustaining scalds and flame burns, should be the primary targets for prevention. Winter was identified as the peak season for pediatric burn injuries. Future burn prevention strategies should therefore be more comprehensive, incorporating burn etiology, regional demographics, natural environmental conditions, socioeconomic status, and education levels to ensure contextual relevance and effectiveness.

In parallel, targeting adult caregivers, must be prioritized as a key component of prevention efforts. Given their central role in daily child supervision and risk management, educational and behavioral interventions aimed at this group are essential to reduce burn incidence in this highly dependent pediatric population. By addressing caregiver knowledge, practices, and environmental awareness, such strategies can create a more protective and sustainable caregiving environment for at-risk children.

## Data Availability

The original contributions presented in the study are included in the article/Supplementary material, further inquiries can be directed to the corresponding authors.
